# Extracellular Vesicle-Associated Transitory Cell Wall Components and Their Impact on the Interaction of Fungi with Host Cells

**DOI:** 10.3389/fmicb.2016.01034

**Published:** 2016-07-08

**Authors:** Leonardo Nimrichter, Marcio M. de Souza, Maurizio Del Poeta, Joshua D. Nosanchuk, Luna Joffe, Patricia de M. Tavares, Marcio L. Rodrigues

**Affiliations:** ^1^Laboratório de Glicobiologia de Eucariotos, Instituto de Microbiologia Professor Paulo de Góes, Universidade Federal do Rio de Janeiro, Rio de JaneiroBrazil; ^2^Department of Molecular Genetics and Microbiology, Stony Brook University, Stony Brook, NYUSA; ^3^Veterans Administration Medical Center, Northport, NYUSA; ^4^Department of Microbiology and Immunology and Medicine, Albert Einstein College of Medicine, Bronx, NYUSA; ^5^Fundação Oswaldo Cruz, Centro de Desenvolvimento Tecnológico em Saúde, Rio de JaneiroBrazil

**Keywords:** fungal cell wall, extracellular vesicles, proteomics, host cell, cell wall remodeling

## Abstract

Classic cell wall components of fungi comprise the polysaccharides glucans and chitin, in association with glycoproteins and pigments. During the last decade, however, system biology approaches clearly demonstrated that the composition of fungal cell walls include atypical molecules historically associated with intracellular or membrane locations. Elucidation of mechanisms by which many fungal molecules are exported to the extracellular space suggested that these atypical components are transitorily located to the cell wall. The presence of extracellular vesicles (EVs) at the fungal cell wall and in culture supernatants of distinct pathogenic species suggested a highly functional mechanism of molecular export in these organisms. Thus, the passage of EVs through fungal cell walls suggests remarkable molecular diversity and, consequently, a potentially variable influence on the host antifungal response. On the basis of information derived from the proteomic characterization of fungal EVs from the yeasts *Cryptoccocus neoformans* and *Candida albicans* and the dimorphic fungi *Histoplasma capsulatum* and *Paracoccidioides brasiliensis*, our manuscript is focused on the clear view that the fungal cell wall is much more complex than previously thought.

## Introduction

Glucans, chitin, and glycoproteins are cross-linked to form the most essential structure of fungal cell walls (Free, 2013). This structure is responsible for cell shaping, as well as for osmotic and physical protection of the cell (Nimrichter et al., 2005). However, fungal morphogenesis and reproduction require elaborated cell wall remodeling. Therefore, the fungal cell wall must combine contrasting properties such as elasticity and rigidity, which demands a remarkable dynamism. In fact, different fungal species have distinct ways to assemble their cell wall (Erwig and Gow, 2016). A plethora of enzymes reach precise regions at the fungal cell surface to finely control remodeling, avoiding cellular damage (Fischer et al., 2008). The consequence of these rearrangements must impact directly on the recognition of fungal pathogens by the host, since they imply a high diversity in the molecular composition of the cell surface.

Several reviews discuss the interaction of fungi with host cells based on well-known surface components, such as α and β-glucans, mannoproteins, galactomannan, glucuronoxylomannan (GXM), and most recently chitin and its derivatives (Romani et al., 2002; Romani, 2011; Vautier et al., 2012; Paulovicova et al., 2014; Dambuza and Brown, 2015; Levitz et al., 2015; Underhill and Pearlman, 2015). However, atypical proteins originally characterized as cytoplasmic or plasma membrane constituents have been also found at cell wall (Alloush et al., 1997; Gil-Navarro et al., 1997; Gozalbo et al., 1998; Pitarch et al., 2002; Kneipp et al., 2003; Motshwene et al., 2003; Nimrichter et al., 2005; Barbosa et al., 2006; Batista et al., 2006; Castillo et al., 2008; da Silva Neto et al., 2009; Tomazett et al., 2010; Brito Wde et al., 2011; Karkowska-Kuleta et al., 2011; Puccia et al., 2011; Marcos et al., 2012; Gil-Bona et al., 2015b; Karkowska-Kuleta and Kozik, 2015). Most of these proteins share a common characteristic: they are released from the cell inside vesicular compartments that traverse the cell wall and reach the extracellular environment. These molecular carriers are called extracellular vesicles (EVs), which are part of a conserved secretion mechanism shared by all domains of life. EV composition, biogenesis, and immunobiological functions were discussed in recent reviews (Rodrigues et al., 2011, 2013, 2014, 2015; Oliveira et al., 2013; Brown et al., 2015) but there remains a significant need for additional information regarding the mechanisms through which EVs pass through the cell wall and how they influence host recognition. In fact, EVs from *Cryptoccocus neoformans* and *Candida albicans* are recognized and internalized by phagocytes culminating in host cell activation (Oliveira et al., 2010; Vargas et al., 2015). Considering the multiplicity in the composition of fungal EVs, a number of receptors are expected to participate in their recognition. For instance, EVs from *Paracoccidioides brasiliensis* carry membrane-bound mannose and *N*-acetylglucosamine and are recognized by DC-SIGN and DC-SIGNR, but not dectin-1 or -2 (Peres da Silva et al., 2015).

In this review we discuss both direct and indirect putative mechanisms of participation of EV-associated molecules during interaction of pathogenic fungi with host cells. In this context, host cell receptors and antibodies could target EV components. Moreover, enzymes carried by these compartments could modify the cell wall and its composition, which might impact cell wall architecture and the pathophysiology of distinct fungal diseases.

## Enzymes From Metabolic Pathways

The mechanisms of EVs biogenesis remains obscure but apparently includes (i) multivesicular body formation followed by exosome release, (ii) vesicle shedding from the plasma membrane, and (iii) inverted macropinocytosis (Rodrigues et al., 2007, 2014, 2015; Vargas et al., 2015). All three mechanisms are in agreement with the compositional complexity of EVs, including membrane and cytoplasmic molecules (Albuquerque et al., 2008; Rodrigues et al., 2008; Vallejo et al., 2011; Wolf et al., 2014; Gil-Bona et al., 2015a; Vargas et al., 2015). Proteomic analyses of fungal EVs from diverse species clearly show a considerable number of enzymes that are associated with metabolic routes (**Table 1**) (Albuquerque et al., 2008; Rodrigues et al., 2008; Vallejo et al., 2011; Vargas et al., 2015). Some of these enzymes are actually conserved among EVs produced by distinct fungal species. Many of them are also considered as moonlighting proteins, implying primary and secondary biological functions (Jeffery, 2014). Major hits include enzymes required for glycolysis, fermentation, gluconeogenesis, pentose phosphate, tricarboxylic acid, and glyoxylate cycles (**Table 1**) (Albuquerque et al., 2008; Rodrigues et al., 2008; Vallejo et al., 2011; Vargas et al., 2015). In this group of molecules, glyceraldehyde-3-phosphate dehydrogenase (GAPDH), enolase, and transaldolase were consistently detected in *C. neoformans*, *C. albicans*, *P. brasiliensis*, and *Histoplasma capsulatum* EVs by proteomic analysis (Rodrigues et al., 2008; Vallejo et al., 2011; Wolf et al., 2014; Gil-Bona et al., 2015a; Vargas et al., 2015). According to Gozalbo et al. (1998) GAPDH, a typical cytoplasmic protein, also decorates the outermost layer of the *C. albicans* cell wall where it mediates adhesion to laminin and fibronectin. The protein, however, is apparently a poor immunogen, since vaccination with GAPDH or exposure of mice to antibodies against GAPDH did not impact the outcome of disseminated candidiasis (Gil et al., 2006). GAPDH was also detected at the cell surface of *P. brasiliensis* yeast forms (Barbosa et al., 2006), where it also promoted binding to fibronectin, laminin, and type I collagen. Adherence and internalization of *P. brasiliensis* yeast forms by pneumocytes was reduced when fungal cells were pretreated with antibodies to *P. brasiliensis* GAPDH or in the presence of the purified enzyme (Barbosa et al., 2006).

**Table 1:** Major proteins characterized in pathogenic fungi extracellular vesicles (EVs) and involved with metabolic routes, cell wall remodeling, and heat shock response.

	*Candida albicans*	*Histoplasma capsulatum*	*Cryptoccocus neoformans*	*Paracoccidioides brasiliensis*
**Glycolysis**				
Hexokinase		+		+
Phosphoglucose isomerase		+		+
Aldolase	+	+		+
Triose phosphate isomerase		+		+
GAPDH	+	+	+	+
Phosphoglycerate kinase	+	+		+
Phosphoglycerate mutase	+			
Enolase	+	+	+	+
Pyruvate kinase	+	+	+	
Aldehyde dehydrogenase	+	+	+	
**Fermentation**				
Alcohol dehydrogenase 2	+	+		
Pyruvate decarboxylase	+	+	+	
**Gluconeogenesis**				
Phosphoglucomutase		+		
Fructose-1,6-bisphosphatase		+		+
Phosphoenolpyruvate carboxykinase		+	+	+
Pyruvate carboxylase	+	+		
**Pentose phosphate**				
Lactonase				
6-Phosphogluconate dehydrogenase	+	+	+	+
Transaldolase	+	+	+	+
Transketolase	+	+	+	+
Triosephosphate isomerase	+	+		+
**Tricarboxylic acid cycle**				
Pyruvate dehydrogenase		+	+	+
Citrate synthase		+	+	+
Aconitase				+
Isocitrate dehydrogenase			+	
α-Ketoglutarate dehydrogenase		+	+	
Succinyl-CoA synthetase		+		
Succinate dehydrogenase		+		
Fumarase		+		
Malate dehydrogenase		+	+	
**Glyoxylate cycle**				
Aconitase		+		
Isocitrate lyase		+		
Malate synthase		+	+	
**Cell wall architecture**				
*Chitinase	+	+		+
β -1,3-glucosyltransferase	+	+	+	
β -1,3-glucan synthase	+			
β -1,6-glucan synthase				
α-1,2-Mannosylphosphate transferase	+			
α-1,3-glucan synthase				+
α-1,3-glucanase			+	
**β -1,3-glucanase	+	+		+
Chitin synthase		+	+	+
Chitin deacetylase			+	
Glycosidase	+	+		
Mannosidase				+
**Heat shock proteins**				
HSP10				+
HSP12	+			
HSP30		+		+
HSP60		+		
HSP70	+	+	+	+
HSP82		+		
HSP88		+		+
HSP90	+		+	+
HSP98				+

*any type of chitinase (secreted or cell wall associated). **endo or exo glucanase.

Enolase is another example of an immunogenic, cytoplasmic protein that actively participates in the fungal-host cell interface. This antigen was detected at large amounts in *C. albicans* supernatants (Sundstrom and Aliaga, 1994). In addition, enolase is one of the main cell wall proteins of *C. albicans* (Angiolella et al., 1996). In fact, enolase is considered the humoral immunodominant antigen in germ free mice and in humans with disseminated candidiasis (Sundstrom et al., 1994; Pitarch et al., 2008). Li et al. (2013) suggested that IgG antibodies against *Candida* enolase and aldolase, in combination, could be markers to diagnose invasive candidiasis. In contrast to GAPDH, anti-enolase antibodies are at least partially protective in murine candidiasis (van Deventer et al., 1996; Montagnoli et al., 2004; Li et al., 2011). Besides its role in the glycolytic pathway and immunogenic properties, enolase participates in host cell adhesion. For instance, the *C. albicans* enzyme recognized plasmin and plasminogen (Jong et al., 2003). Furthermore, plasmin-bound yeast cells displayed an improved ability to induce fibrinolysis in a matrix-gel assay as well as to cross an *in vitro* blood brain barrier system. Likewise, enolase can participate during *C. albicans* intestinal colonization. Yeast adhesion to the intestinal epithelium was inhibited by enolase containing-disks or by pretreatment with antibodies to enolase (Silva et al., 2014). Similarly, adhesion of *P. brasiliensis* to host cells and fibronectin required enolase, which is also cell wall-bound in this fungus (Nogueira et al., 2010; Marcos et al., 2012). The relevance of enolase during infection was confirmed by the demonstration that its expression was upregulated in yeast cells of *P. brasiliensis* recovered from infected mice tissues (Nogueira et al., 2010). In addition, enolase and GADPH association with plasminogen resulted in plasmin formation through tissue plasminogen activator in a lysine dependent fashion. As a consequence fibronectin was degraded on the fungus surface. Recently, enolase was also detected at cell surface of *A. fumigatus*, *A. flavus*, *A. terreus*, *A. nidulans*, and *C. glabrata* (Funk et al., 2016). As observed for other fungal species, enolase from *A. fumigatus* binds to plasminogen remaining accessible to plasminogen activator uPA, which confirms its potential to participate during fungal dissemination (Funk et al., 2016).

Additional metabolism-related enzymes have been associated with host cell recognition. Phosphoglycerate mutase 1 (Pgmt1) from *C. albicans* binds to factor H, FHL-1 and plasminogen (Crowe et al., 2003; Poltermann et al., 2007). Lopez et al. (2014) also demonstrated that Pgmt1 interacted with fibronectin and vitronectin. Pgmt1 is recognized by human umbilical vein endothelial cells (HUVEC), keratinocytes (HaCaT cells), and U937 monocytic cells (Lopez et al., 2014). Consistent with these results, *C. albicans* mutants in which the enzyme was knocked out displayed a reduced capacity to bind HUVECS. Thus, Pgmt1 appears to be linked to fungal pathogenesis by activating the factor H, FHL-1, and plasminogen for immune evasion and degradation of extracellular matrices. The notion that metabolic enzymes in fact affect fungal pathogenesis was confirmed by results with triosephosphate isomerase (Tpi). This enzyme was also found at cell wall of *P. brasiliensis* and binds to laminin (Pereira et al., 2007). The use of polyclonal antibodies to Tpi inhibited the interaction of yeast with epithelial cells *in vitro*, suggesting that it also intermediates the association with host cells.

The enzymes mentioned above and others can operate through integrated mechanisms. Crowe et al. (2003) reported at least eight plasminogen-binding proteins at the cell wall of *C. albicans* (Crowe et al., 2003). Six of them were detected in EVs produced by *C. albicans*, including the enzymes Pgmt1, alcohol dehydrogenase, GAPDH, phosphoglycerate kinase, and aldolase (Gil-Bona et al., 2015a; Vargas et al., 2015). These proteins were associated with the capacity of *C. albicans* to activate plasminogen, resulting in more effective tissue invasion (Crowe et al., 2003).

The combination of proteins exported in EVs could influence recognition of other fungal species by host cells. Fibronectin, vitronectin and laminin recognize cytoplasmic proteins that are surface-exposed in *C. parapsilosis* and *C. tropicalis* pseudohyphae, including malate synthase, glucose-6-phosphate isomerase, 6-phosphogluconate dehydrogenase, enolase, fructose-1,6-bisphosphatase, transketolase, transaldolase, and elongation factor 2 (Kozik et al., 2015). In *C. neoformans*, phosphoglycerate kinase, transaldolase, aldolase, and glutamate dehydrogenase demonstrated the capacity to bind plasminogen (Stie et al., 2009). As shown in other species, surface-bound active plasmin in *C. neoformans* increased the ability of the fungus to penetrate the brain.

Association with extracellular matrix proteins and activation of plasmin are not the only potential activities of glycolytic enzymes in *C. albicans*. Karkowska-Kuleta et al. (2011) showed that enolase, Tgpm1, and Tpi are able to bind kininogen culminating with kinin activation (Karkowska-Kuleta et al., 2011). These studies support the hypothesis that fungal EVs correspond to antigen-rich compartments responsible for the delivery of metabolic enzymes interfering with host’s physiology.

## Heat Shock Proteins

Similar to what is detailed for the above glycolytic enzymes, HSP70 is carried by fungal EVs through the cell wall (Albuquerque et al., 2008; Rodrigues et al., 2008; Vallejo et al., 2011; Wolf et al., 2014; Gil-Bona et al., 2015a; Vargas et al., 2015). Its participation during interaction with host cells has been investigated in *C. neoformans* and *C. albicans*. In the former, HSP70 is present at the fungal surface, within the capsular network (Silveira et al., 2013). Recombinant HSP70 (Cn-rHSP70) from *C. neoformans* is efficiently internalized by the macrophage like-cell line J774.1 and, to a minor extent, by A549 pneumocytes. Pre-treatment of J774.1 cells with Cn-rHSP70 does not impair phagocytosis, but increases fungal survival within macrophages accompanied by a decrease in nitric oxide (NO) production. In addition Cn-rHSP70 can upregulate TLR4 expression in macrophages (Silveira et al., 2013) and directly interfere with early macrophage polarization, limiting innate control of *C. neoformans* (Eastman et al., 2015). These results indicate that EVs carry proteins that facilitate *C. neoformans* survival within the host.

*Candida albicans* expresses two major HSP70 proteins, SSA1 and SSA2 (Lopez-Ribot et al., 1996). SSA2 has been immunolocalized at the plasma membrane and cell wall in both yeast and hyphal forms (Lopez-Ribot et al., 1996). The protein is recognized by histatin 5, a member of the family of small histidine-rich antifungal peptides secreted by salivary glands (Li et al., 2003), leading to its internalization and consequent fungal death (Li et al., 2006). On the other hand SSA1 is required for endocytosis by endothelial and epithelial cells *in vitro* through a cadherin-dependent recognition mechanism (Sun et al., 2010). Furthermore, SSA1 appears to act as an invasin contributing to *C. albicans* virulence in hematogenously disseminated and oropharyngeal candidiasis (Sun et al., 2010). In addition, SSA1 contributes at least partially to *C. albicans* penetration to microfold-like cells generated by the co-culture of enterocytes with B lymphocytes (Albac et al., 2016). Thus, proteins carried by *C. albicans* EVs can have opposite effects when in contact with host cells.

Vesicles from *H. capsulatum* carry distinct heat shock proteins (Albuquerque et al., 2008). HSP60 is one of the major hits in *H. capsulatum* EVs. A series of studies investigating binding and internalization of *H. capsulatum* yeasts by macrophages revealed a key function for this protein, which accumulates at the fungal cell wall (Long et al., 2003). HSP60 from *H. capsulatum* is recognized by the integrin CD18, a CR3 subunit at the macrophage cell surface (Long et al., 2003; Habich et al., 2006). Through this association yeasts of *H. capsulatum* are internalized and evade the macrophage defense (Strasser et al., 1999; Woods, 2003). In addition, HSP60 is considered an immunodominant antigen that orchestrates the adaptation to temperature stress (Deepe and Gibbons, 2002; Scheckelhoff and Deepe, 2002; Guimaraes et al., 2011b). Immunization of mice with recombinant HSP60 induces a protective response against *H. capsulatum* (Deepe and Gibbons, 2002; Scheckelhoff and Deepe, 2002). Furthermore, passive administration of IgG1 and IgG2a against HSP60 in a murine model of histoplasmosis promotes a protective effect associated with higher levels of IL-2, IL-12, and IFN-γ and decreased levels of IL-4 and IL-10 (Guimaraes et al., 2009). Different independent mechanisms could be linked to the antibody effect *in vivo*. First, antibodies to HSP60 alter the rates of phagocytosis and killing of *H. capsulatum* yeast cells by host effector cells (Guimaraes et al., 2009) as well as cause agglutination of *H. capsulatum* yeasts, which further alters interactions with macrophages and induces changes in macrophage antifungal functions (Guimaraes et al., 2011a). Recently, we demonstrated that *H. capsulatum* yeasts exposed to antibodies to HSP60 release EVs with different sizes and altered protein loads, including varying the quantity of virulence-associated products, when compared to untreated controls, which suggests that antibodies alter fungal susceptibility to host defenses (Baltazar et al., 2016). In this scenario, HSP60 emerges as an interesting target for the development of new therapies against *H. capsulatum*.

## Polysaccharide Hydrolases

**FIGURE 1:**
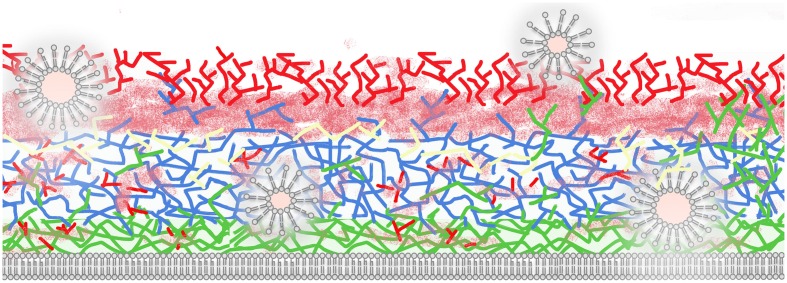
**Schematic illustration of a fungal cell wall and its major polysaccharides and proteins (based on species with protein EV composition characterized).** Extracellular vesicles (EVs) are shown as bilayered compartments. Structural polysaccharides include chitin (green, close to plasma membrane), β1,3 (blue), and β1,6 glucans (yellow). Chitin oligomers (green, distributed across the cell wall), mannans, and mannoproteins (solid and fuzzy red) are also illustrated. EVs traverse the cell wall potentially promoting remodeling through hydrolysis of polysaccharides and mannoproteins and exposing internal structural components to the extracellular environment. For didactic purposes, melanin, lipids, capsule, β 1,4, and α 1,4 glucans and other cell wall components are not illustrated in this model.

As mentioned previously, the complexity of the cell wall requires highly coordinated mechanisms to allow morphological rearrangements supporting fungal growth, budding and hyphal formation. In addition, cell wall composition can be robustly modified according to the species and growth conditions (Erwig and Gow, 2016). **Figure 1** shows a simplified general picture of the fungal cell wall in which layers of chitin are displayed adjacent to the cell membrane, although oligomers of chitin have been observed in other regions of the fungal surface (Fonseca et al., 2009). The main chitin layers are connected to a glucan network that can include β1,3, β1,4, β1,6, and α1,3 linkages (Free, 2013; Erwig and Gow, 2016). Proteins are associated to the cell wall (cell wall proteins, CWP) through both covalent and non-covalent bonds (Chaffin, 2008; Heilmann et al., 2012; Orlean, 2012). At least three types of proteins can be covalently linked to fungal polysaccharides: (i) proteins with an alkali-sensitive linkage (ASL), which are linked to β1,3 glucans, (ii) proteins covalently bound to β-1,6-glucan via a remnant of a glycosylphosphatidylinositol (GPI) anchor, and (iii) proteins linked to wall polysaccharides through disulfide bonds. Non-covalently bound proteins encompass transitory polypeptides that are synthesized intracellularly and targeted for extracellular secretion (Chaffin, 2008).

Environmental changes are associated with cell wall remodeling, including nutrient availability, pH and temperature (Sosinska et al., 2008; Heilmann et al., 2013; Ene et al., 2015). In a recent study, Ene et al. (2015) demonstrated substantial cell wall transformation after only thirty seconds in response to hyperosmotic stress. They showed changes in cell wall volume can be accompanied by ultrastructural adjustments, including (i) increase of the inner β-glucan and chitin layers and (ii) contraction of the mannoprotein layer. These drastic modifications require enzymatic activities of synthesis and degradation. To hydrolyze structural components, chitinases, mannosidases, and glucosidases (glucanases) are mandatory. In fungal EVs a number of hydrolases, including glycosidases, lipases, and proteases, were characterized (Rodrigues et al., 2007, 2008; Albuquerque et al., 2008; Vallejo et al., 2011; Gil-Bona et al., 2015a; Vargas et al., 2015). These compartments could be responsible for prompt changes at cell wall by releasing pre-formed enzymes during a stress response.

Several host cellular receptors for fungal cell wall polysaccharides are reported in the literature, including CR3 (CD18/CD11b), Toll like receptors (TLRs), Dectin 1 and 2, DC-SIGN, mannose receptors, CD14, lactosylceramide, and Mincle (for details, see Zimmerman et al., 1998; Kimberg and Brown, 2008; Barreto-Bergter and Figueiredo, 2014; Dalonso et al., 2015). Although functional studies of polysaccharide recognition have traditionally focused on typical cell wall components, it is important to mention the products of polysaccharide hydrolases have similar potential to be recognized by receptors and immunologically active. For instance, in the *C. neoformans* model, chitooligomers released through chitinase activity form soluble complexes with capsular GXM, resulting in hybrid glycans with unique immunological activity (Ramos et al., 2012). Enzymatically released oligosaccharides and (glyco)proteins could also modify the extracellular microenvironment and potentially impact the immune response, by scavenging antibodies and carbohydrate binding proteins (CBPs). Finally, cell wall components can also suffer modifications derived from the activity of host hydrolases. In *C. neoformans*, chitin cleavage via chitotriosidase promoted pathologic type-2 helper T cell responses (Wiesner et al., 2015).

The level of glucanase activities at the cell wall can potentially influence fungal recognition, finally interfering with receptor-ligand connections. For instance, along with CR3 (CD18/CD11b), dectin-1 is a major ligand responsible for β1,3 glucan cell wall detection culminating with phagocytosis, oxidative burst response and cytokine production (Brown and Gordon, 2001; Huang et al., 2015). Host cells that express dectin-1 include monocytes, macrophage, neutrophils, and dendritic cells (Taylor et al., 2002; Willment et al., 2005). Li et al. (2012) showed that treatment of *C. albicans* with β1,3 glucanase abolished fungal recognition through dectin-1 by neutrophils. Although some authors have shown that either soluble and particles of β1,3 glucans modulate the function of host cells (Drummond and Brown, 2011), studies by Goodridge et al. (2011) suggested that signaling is triggered only after dectin-1 binding to particulate β-glucans. The fact that particles of β1,3 glucans have a higher valence for dectin-1 recognition and consequent enhanced efficacy in the induction of cross-talks between other ligands, including CR3 and TLRs (O’Neill, 2008), suggests that the stimulatory mechanisms triggered by soluble and particle β1,3 glucans must be in fact distinct. The major β1,3 glucanases characterized in *C. albicans* EVs were Xog1p, Eng1, Sun41 and MP65 (Gil-Bona et al., 2015a; Vargas et al., 2015). Xog1p is the major β-1,3-exoglucanase in *C. albicans* (Gonzalez et al., 1997) that is a receptor for the antimicrobial peptide LL-37, produced by human neutrophils (Turner et al., 1998; Tsai et al., 2011a). LL-37 kills *C. albicans* and, in addition, reduces binding of *C. albicans* to plastic surfaces, oral epidermoid OECM-1 cells, and murine urinary bladders at sub-inhibitory concentrations (Tsai et al., 2011b). Additional ligands to LL-37 include mannans, glucans, and chitin (Tsai et al., 2011b). The mechanisms involved in inhibition of cell adhesion include direct competition and interference with glucanase activities leading to disturbance of cell wall remodeling (Tsai et al., 2011b; Chang et al., 2012). The putative glucosidase SUN41 and MP65 are also exported in *C. albicans* EVs (Gil-Bona et al., 2015a; Vargas et al., 2015). SUN41 is regularly involved with cytokinesis, cell wall biogenesis, adhesion to host tissue, and biofilm formation (Hiller et al., 2007).

MP65 is a major immunogenic mannoprotein secreted by *C. albicans* and other *Candida* species (Gomez et al., 1996; Karkowska-Kuleta et al., 2015). MP65 is found at the cell wall and its secretion occurs potentially due to the presence of a Kex2 site (Newport et al., 2003). A protective response generated after vaccination with a low-virulence *Candida* strain was associated with cell-mediated immunity disclosed by MP65 stimulation of splenocytes *in vitro* and a delayed-type hypersensitivity response *in vivo* (Mencacci et al., 1994; Cassone et al., 1998). Human DCs were stimulated by MP65 culminating with TNF-α and IL-6 release and the activation of IL-12 expression. Maturation of DCs was confirmed by increasing co-stimulatory molecules such as CD40, CD80, CD86, MHC class II, and decreasing CD16, CD32, and CD64 (Pietrella et al., 2006). Recombinant MP65 was also internalized by macrophages and DCs in a mechanism at least partially associated with a RGD peptide sequence (Pietrella et al., 2008). However, differently from the native mannoprotein, TNF-α and IL-6 were not induced by recombinant MP65 (Pietrella et al., 2008). These data suggested that cytokine production was potentially stimulated by the glycan moiety of MP65, probably through lectin and TLR-dependent mechanisms, as suggested by Netea et al. (2006). However, both cells were able to stimulate T-cell activation with IFNγ and IL-4 production, confirming the potential activity of the protein sequence of MP65 (Pietrella et al., 2008). In *C. albicans*, the activity of β1,3 glucanase also appears to be involved with yeast filamentation at 37°C, which also supports its relevance during infection progress (Xu et al., 2013). Influence of other glucanases exported in *C. albicans*, *H. capsulatum*, *C. neoformans*, and *P. brasiliensis* EVs during host-cell recognition has not been reported in the literature; however, as mentioned previously, their activities as cell wall remodeling enzymes could impact the distribution of native proteins, polysaccharides, and their products of hydrolysis, consequently modulating the immune response.

## Concluding Remarks

The current literature shows that the fungal cell wall composition is highly complex and varies considerably according to the species investigated. The basic cell wall network is composed by structural components covalently connected to each other. Thus, molecular changes at this level require an intense participation of hydrolytic enzymes and transient molecules. Furthermore, such modifications interfere significantly with the way a fungal pathogen is coated and directly influence its engagement with a host cell. Based on the recent literature we believe that the complexity of the fungal cell wall could be significantly impacted by the presence and passage of EVs. The presence of enzymes and virulence regulators characterized in EVs produced by four distinct major pathogens suggests that these compartments could tailor the cell wall supporting significant changes in short periods of time. The ability of other medically relevant fungal species, including molds such as *Aspergillus sp*, to release EVs is still under investigation. In fact, typical cytoplasmic and membrane proteins from *A. fumigatus* were detected at the cell wall (Champer et al., 2016), supporting the hypothesis that they are trafficked in EVs. In this sense, hexokinase, aldolase, phosphoglycerate mutase, β1,3 glucosyltransferase, chitinase, mannosidase, β1,3 glucanase, among others, were characterized at cell wall extracts from *A. fumigatus* (Champer et al., 2016). In addition, enolase, transaldolase, β1,3 glucosyltransferase, β1,3 glucanase, α1,3 glucan synthase, α1,3 glucanase, chitinase, mannosidase were detected in the *A. fumigatus* secretome (Adav et al., 2015; Champer et al., 2016).

Through these heterogeneous compartments a number of proteins reach the fungal cell surface, consequently modifying cell wall composition and affecting fungal-host cell interactions. EVs can also release immunoactive compounds to the extracellular environment, impacting fungal pathogenesis. Consequently, biogenesis of EVs is a potential target for the development of novel antifungal drugs. In addition, the diversity of native immunogenic proteins carried by EVs suggests that these compartments are multi-antigen platforms that could be used in vaccine formulations.

## Author Contributions

All authors listed, have made substantial, direct, and intellectual contribution to the work, and approved it for publication.

## Conflict of Interest Statement

The authors declare that the research was conducted in the absence of any commercial or financial relationships that could be construed as a potential conflict of interest.
